# Antibiotic residues and microbial contamination in pasteurized whole milk intended for human consumption

**DOI:** 10.14202/vetworld.2024.720-727

**Published:** 2024-03-25

**Authors:** Juan Londoño-Carmona, Sandra Blandón-Escobar, John Montoya-Zuluaga, Patricia Betancourt-Chaves, Sara Castillo-Moreno, Carlos Arboleda-Múnera, Darío Vallejo-Timarán

**Affiliations:** 1 Center of Natural and Renewable Resources, La Salada. National Learning Service – SENA, Research Group La Salada, Government of Colombia. Caldas, Colombia; 2 Veterinary Medicine and Animal Sciences Faculty, Research Group GIsCA, University Vision de las Americas, Medellín, Colombia; 3 Colombian Agricultural Research Corporation – AGROSAVIA. Obonuco Research Center, Pasto, Colombia

**Keywords:** biological contamination, cow milk, drug residues, food safety

## Abstract

**Background and Aim:**

Milk contamination for human consumption is one of the biggest concerns worldwide. To prevent milk contamination, it is important to implement sustainable production practices that ensure animal health and guarantee veterinary drugs have been used properly. This study aimed to detect antibiotic residues and microbial contamination in commercially available pasteurized whole milk intended for human consumption.

**Materials and Methods:**

We conducted a cross-sectional study on all brands of pasteurized milk (n = 17) for human consumption in Medellín, Colombia, from February 30 to April 30, 2022. Six milk samples of each brand were collected every 15 days, resulting in 102 samples. IDEXX SNAPduo™ ST Plus test (IDEXX Laboratories Inc, Maine, USA) was used to detect cephalosporins residues to detect beta-lactam and tetracyclines. We detected mesophilic aerobic bacteria and coliforms using Chromocult Coliform Agar^®^ (Merck KGaA, Darmstadt, Germany) and Plate-Count Agar^®^ (Merck KGaA), respectively.

**Results:**

Beta-lactam residues were found in 24.4% of the brands. No tetracyclines or cephalosporins were detected. Mesophilic aerobic bacteria and coliform contamination were detected in 42.6% and 12.8% of the brands, respectively. No fecal coliform contamination was detected.

**Conclusion:**

This study demonstrated the presence of antibiotic residues and microbial contamination in commercially available pasteurized whole milk intended for human consumption in the study area, highlighting its potential public health implications.

## Introduction

Milk production is an important economic activity worldwide. According to the Food and Agriculture Organization of the United Nations, in 2020, more than 853 million tons of milk were produced worldwide, with the main producers being the European Union, India, and the United States [1]. Milk production is expected to be an important source of income for farmers because dairy products are one of the main agricultural products produced in many countries [2]. As the global demand for dairy products continues to increase, milk production is expected to remain an important industry in the future [1, 2]. In Colombia, the milk industry has significant economic and social importance. The dairy industry accounts for 2.3% of Colombia’s gross domestic product (GDP) and 24.3% of its agricultural GDP, making it one of the most important economic sectors in the country [3]. Colombia accounts for more than 321,000 milk producers in 22 of 32 regions of the country. In the northern part of the region, Antioquia is the main dairy area, followed by Cundinamarca, where 18.0% of the national production is concentrated [3, 4]. In Colombia, the average milk production per cow is approximately 3.9 L/day [4]. In addition, the consumption of milk and dairy products is high in Colombia, with an average of 150 L of milk/person/year [3].

In Colombia and around the world, dairy production is closely linked to food safety; therefore, it is important to ensure that it is suitable for human consumption. To achieve this objective, it is necessary to comply with quality requirements and standards throughout the milk production process, starting from the feeding of cattle to the marketing of final products. The Food and Agriculture Organization recommends adequate hygiene in all processes and storage, the implementation of quality control programs, the adoption of a risk-based food safety approach, and the identification of critical control points [1]. In addition, sustainable production practices must be implemented to ensure animal health and welfare and to ensure the proper and responsible use of veterinary drugs to prevent milk contamination [5]. Contamination of human milk consumption is one of the biggest concerns worldwide because of the presence of microbial and antibiotic residues in milk [5]. Milk may contain contaminants, such as pathogenic bacteria such as *Listeria monocytogenes*, *Salmonella* spp., *Escherichia coli*, and *Staphylococcus aureus*, as well as residues of antibiotics, pesticides, and other chemicals, which can be harmful to human health [6]. Penicillin, tetracycline, sulfonamides, cephalosporins, and streptomycin, which can be a risk to human health due to the development of antibiotic resistance [7], are the main antibiotics found in milk. Residues and contaminants in dairy products for final consumption have previously been reported in other countries [6–13]. The National Institute for Food and Drug Surveillance (INVIMA) of the Colombian Government regulates and controls drugs and other food contaminants in Colombia. However, limited information is available in the country on the hygiene and hygiene quality of certain products intended for final consumption, such as pasteurized whole milk.

This study aimed to detect antibiotic residues and microbial contamination in commercially available pasteurized whole milk intended for human consumption in Medellín, Colombia.

## Materials and Methods

### Ethical approval

The study was conducted following approved experimental practices and standards by the Institutional Committee for the Care and Use of Animals - CICUA, University Vision de las Americas, Colombia (Ethical approval code 036, August 11, 2021).

### Study period and location

The study was conducted from February 30 to April 30, 2022, in the Technological Services Laboratory, Microbiology Section, La Salada, Center for Renewable Natural Resources (SENA), located in the municipality of Caldas Antioquia, Colombia.

### Population analysis and experimental design

This was a cross-sectional study of pasteurized milk brands marketed for human consumption in Medellín, Colombia. The milk brands were purposively selected based on the following criteria: whole pasteurized milk only and brands that reported their composition. A total of 17 milk brands were selected.

### Sampling and data collection

**Figure-1:**
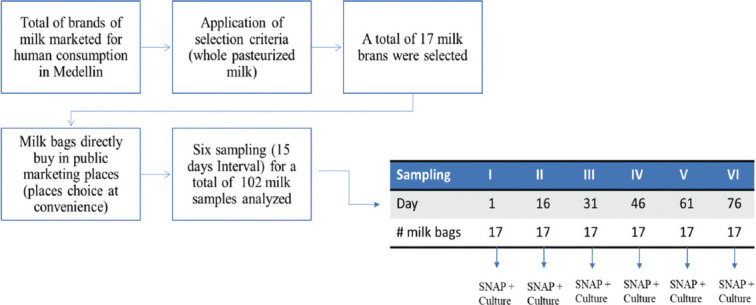
Experimental schedule for the determination of antibiotic residues, aerobic mesophilic, and coliform bacteria detected in pasteurized whole milk marketed for human consumption.

Six samples were collected from each of the selected brands (n = 17) at a 15-day interval (Figure-1) [14]. A total of 102 milk samples were analyzed to detect antibiotic residues and identify microorganisms. Each brand’s milk bags were bought directly in public marketing places; these places were chosen at convenience based on the availability of the selected brands and their localization in Medellín, Colombia. The milk bags were stored at an average temperature of 6°C and transported to the microbiology laboratory of the National Learning Service (SENA), La Salada, Antioquia, Colombia. To maintain a constant refrigeration temperature during the obtention, transport, and sampling, the samples were carefully followed.

**Figure-2:**
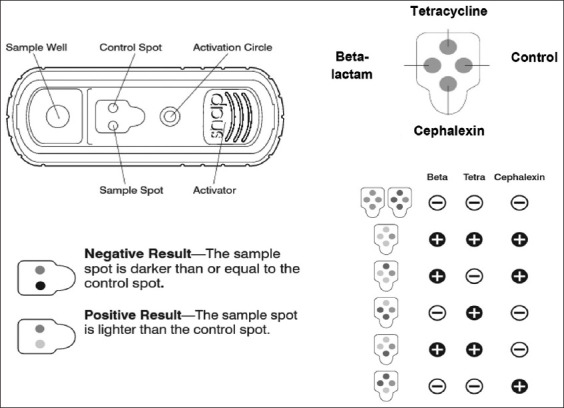
IDEXX SNAP duo™ ST plus test – results interpretation [14].

Samples (n = 102) were analyzed using the IDEXX SNAPduo™ ST Plus Test (Beta-Lactam and Tetracycline), IDDEXX Laboratories Inc., Maine, USA, to detect antibiotic residues. This kit is a screening test validated for the detection of beta-lactams (including cephalexin) and tetracycline residues in cow milk. For the test, the sample was extracted using a kit pipette, and milk was transferred into the kit tube up to the indicator line (450 μL ± 50 μL of milk). The tube was gently shaken to dissolve the conjugate tablet, and the sample was poured into the SNAP device well. When the sample reached the activation circle, the device was pressed until the SNAP sound was heard, and the results were read at 6 min. Positive results were defined according to the kit recommendations (Figure-2).

Chromocult Coliform Agar^®^ (Merck KGaA, Darmstadt, Germany) and Plate-Count Agar^®^ (Merck KGaA, Darmstadt, Germany) were used to detect aerobic mesophilic and coliform bacteria. Three milk/peptone water dilutions (1/10 dilution, 2/10 dilution, and 3/10 dilution) were used. Each dilution was placed in a sterile Petri dish and inoculated with 1 mL of the dilution; the Chromocult^®^ or Plate-Count^®^ (Merck KGaA) culture medium was added as appropriate, gently shaken, and allowed to solidify. Both culture media were incubated at 35°C ± 2 for 48 h. Colony and coliform counts were performed using Plate-Count Agar® and Chromocult Agar^®^ (Merck KGaA). For both mesophiles and coliforms, contamination was defined as a major cutoff value of 10 colony-forming units (CFU) per mL of sample (>10 CFU/mL).

### Statistical analysis

The study variables were as follows: (1) Antibiotic residual variables: (a) Presence/absence of beta-lactam and (b) presence/absence of tetracycline. (2) Milk bacteria variables: (a) Aerobic mesophiles >10 CFU/mL (yes/no); (b) total coliforms >10 CFU/mL (yes/no); and (c) fecal coliforms >10 CFU/mL (yes/no); and (3) brand variables: (a) Brand and (b) distribution of the milk brand (regional/national) and sampling period (I–VI).

Each variable was statistically analyzed using descriptive statistics by frequency tables, estimating the percentage of each variable in the total sample. The distribution of antibiotic residues and milk bacteria variables in relation to milk brand variables and the sampling period were also analyzed, and differences were estimated using a non-parametric method. Statistical variables were analyzed using STATA™ Statistical Software, Version 18.0 (Stata Corp., Dallas, TX, USA).

## Results

### Aerobic mesophilic and coliform bacterial contamination in pasteurized whole milk

**Figure-3:**
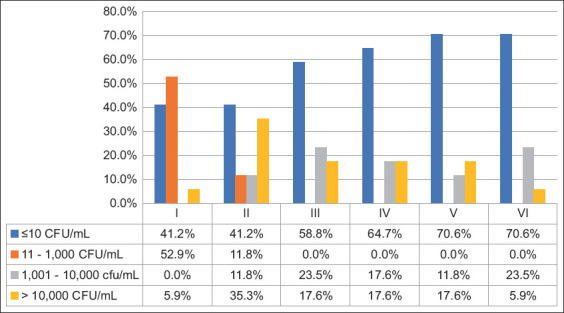
Detection (CFU/mL) of aerobic mesophilic bacteria (APC) in samples (n = 102) from 17 pasteurized whole milk brands marketed for human consumption, collected each 15-day for 90 days (sampling I–VI) in the city of Medellín, Colombia. CFU=Colony-forming units.

**Figure-4:**
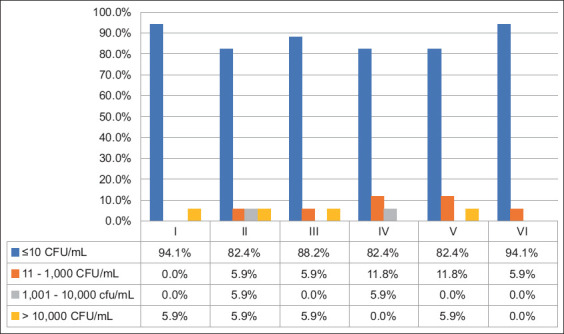
Detection (CFU/mL) of coliform bacteria in samples (n = 102) from 17 pasteurized whole milk brands marketed for human consumption, collected each 15-day for 90 days (sampling I–VI) in the city of Medellín, Colombia. CFU=Colony-forming units.

Aerobic mesophilic bacteria, total coliforms, and *E. coli* were found in 42.6%, 12.8%, and 0.0% of the samples, respectively. Aerobic mesophilic bacteria (Figure-3) varied from 11 to 1,000 CFU/mL (10.9%), 1,001 to 10,000 CFU/mL (14.8%), and more than 10,000 CFU/mL (16.8%). Of the brands, 41% had a mesophile count >10,000 CFU/mL during all sampling times, mainly in time II, where 35.0% of the samples had mesophile counts >10,000 CFU/mL. The total number of coliform bacteria (Figure-4) ranged from 11 to 1000 CFU/mL (6.9%), 1001 to 10,000 CFU/mL (2.0%), and more than 10,000 CFU/mL (3.9%). A total coliform count exceeding 10,000 CFU/mL was observed in the culture of a brand (5.8%) during all sampling times.

### Antibiotic residues present in pasteurized whole milk

**Figure-5:**
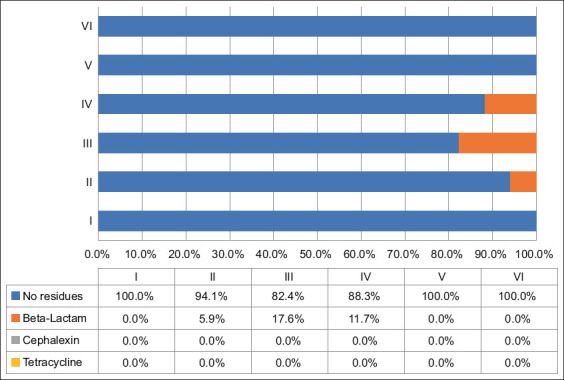
Detection of antibiotic residues in samples (n = 102) from 17 pasteurized whole milk brands marketed for human consumption, collected each 15-day for 90 days (sampling I–VI) in the city of Medellín, Colombia.

Milk samples (n = 102) analyzed using the SNAP kit showed positive results for beta-lactam residues in 5.9% (n = 6) of the samples. Tetracycline and cephalexin residues were not positive. Figure-5 shows the distribution of antibiotic residues in pasteurized whole milk by brand and sampling. Beta-lactam residues were found in 29.4% (n = 5) of the total brands analyzed (n = 17).

## Discussion

### Aerobic mesophilic bacteria in pasteurized whole milk

According to the results, 17.64% of the analyzed brands yielded an aerobic plate count (APC) >80,000 CFU/mL (average APC 670,625 CFU/mL). In 11.76% of the brands, there was a high APC in more than two sampling periods. According to Colombian regulations (Decree Law 616 of 2006), the APC values reported in the present study are higher than the accepted values. APC must be below 40,000 CFU/mL for high-quality pasteurized milk and below 80,000 CFU/mL for acceptable-quality pasteurized milk [14]. The results of this study suggest that the consumption of pasteurized whole milk in Medellín, Colombia, poses a significant risk to public health. Although 82.4% of the brands in the present study reported an acceptable range of APC (between 10 and 40,000 CFU/mL) according to Colombian regulations to identify good quality pasteurized milk, these results indicate that the safety of milk production and processing needs to be carefully evaluated and monitored in the region to reduce the risk of health effects. These findings have important implications for policymakers, public health professionals, and the dairy industry to prevent the marketing of hygienic quality dairy products. Our results highlight the need to increase the surveillance and control of the milking process not only by government regulatory entities but also by milk processing companies. Contrary to international regulations, the Codex Alimentarius [15] states that the maximum limit of APC in pasteurized milk should be 20,000 CFU/mL for warm countries and <100,000 CFU/mL for cold countries. In the United States, the Food and Drug Administration (FDA) sets an upper limit of APC of 10,000 CFU/mL [15]. In the European Union, the maximum permissible limit is 50,000 CFU/mL [15]. In Australia and New Zealand, the maximum allowable limit is 5000 CFU/mL, whereas in Canada, the maximum allowable limit is 30,000 CFU/mL [15]. It should be noted that these limits may vary depending on the regulations in each country [16]. However, our findings indicate that some brands exceeded the APC limit set by international regulations in at least one sample period.

The poor microbiological quality of raw milk may lead to a high concentration of bacteria due to inadequate milking practices and storage at temperatures above 7°C. Milk contains a wide range of nutrients, vitamins, proteins, fats, and carbohydrates that serve as a culture medium for many microorganisms [17]. On the other hand, high APC counts in pasteurized milk could indicate bacterial survival (thermoduric bacteria) during pasteurization. Although mastitis-associated bacteria are not thermoduric, their survival in bulk tank raw milk has been reported [18], which could explain our findings. Pasteurization effectively controls microorganisms that can cause milk quality issues and health risks to consumers [19]. However, changes in store temperature and refrigeration period are related to an increase in bacterial activity up to 15 times [20]. As the temperature increases, bacterial activity increases exponentially, deteriorating the quality of milk in <24 h. This temperature change is more significant in tropical countries such as Colombia, where the average annual temperature ranges from 19°C to 30°C [20]. Other factors, such as lactation status and cow diseases, can increase the bacterial load and deteriorate the sanitary state of milk [21].

APC is an indicator of the hygienic quality of milk. APC indicates the presence of microorganisms that grow in aerobic conditions and may be present in milk due to contamination by the environment, milk industry utensils, or animals [22]. Milk with a high APC may indicate poor hygiene during milking and milk processing, which is a public health risk [23]. In addition, mesophiles can produce enzymes and acids that may affect the taste of milk. To ensure food safety, it is necessary to implement comprehensive policies aimed at monitoring the dairy industry throughout the entire production chain, from the farm and milking process to the final product for consumption.

### Coliform bacteria present in pasteurized whole milk

The presence of fecal and total coliforms is an indicator of sanitary quality of milk, which is important to reduce the risk to human health related to food consumption. Coliform bacteria are present in the environment and in the intestinal tract of animals; the presence of coliform bacteria in milk may indicate fecal or environmental contamination. Fecal coliforms indicate fecal contamination related to the presence of pathogens (e.g., *Salmonella* spp. and *E. coli* O157: H7) in milk, which can cause illnesses in consumers [24]. Although total coliforms include fecal coliforms, they also consist of other non-pathogenic human species [25, 26]. The presence of *E. coli* was not detected throughout the entire sampling period; however, the average total coliform count was found to be 1094.9 CFU/mL for all milk brands throughout the sampling period. In Colombia, government regulations on milk quality specify that the permissible level of total coliforms for pasteurized whole milk must be <10 CFU/mL [27].

The study included all brands of pasteurized whole milk marketed for human consumption in Medellín, Colombia, with more than four million inhabitants [28]. Our report indicates that 17.7% of milk brands in Colombia exceeded the permissible limit of coliforms (CFU/mL), with values exceeding 10 CFU/mL throughout the sampling period. Nevertheless, 35.3% of the brands exceeded permissible coliform levels of the national regulations, with values ranging from 11 to 1,000 CFU/mL in at least one sampling period. Surprisingly, during at least one sampling period, coliform levels ranged from 1001 to 10,000 CFU/mL or exceeded 10,000 CFU/mL in 47.0% of the marketed brands in the city. In Brazil, 52.0% of pasteurized milk samples and 85.0% of raw milk samples were found to be contaminated with fecal and total coliforms [10]. Another study in Brazil reported that 70.8% of milk samples did not comply with current legal regulations for total coliforms in milk and that contamination with coliforms in pasteurized milk was associated with factors related to pre- or post-thermal treatment steps [29]. In Venezuela, 45.0% of the samples exceeded the allowed limit for total coliforms according to the norm, and 72.0% exceeded the acceptance limit for thermotolerant coliforms [30]. In the same country, 50.9% of pasteurized milk samples 1.0 × 10² CFU/mL for coliforms, 5.9 × 10³ CFU/mL for thermophilic bacteria, and 8.7 × 10² CFU/mL for psychrotrophic bacteria [31].

As a result, higher total coliform counts indicate failure of pasteurization process (not sufficient to eliminate milk bacteria) or poor hygiene during milk production, transportation, or storage.

### Antibiotic residues

Consumption of small amounts of antibiotic residues in milk can contribute to the development of long-term antibiotic resistance [32]. Furthermore, it has been reported that resistance genes are transferred from antibiotic-resistant bacteria in animals to bacteria in humans through food [33, 34]. A correlation has been found between the consumption of food contaminated with antibiotic residues and the emergence of antibiotic-resistant strains in humans [35]. To prevent the spread of antibiotic resistance among people, it is important to mitigate the presence of antibiotic residues in food. In addition, people who are allergic to antibiotics may experience allergic reactions when they consume milk containing antibiotic residues.

In contrast to our results, studies in South America found antibiotic residues in milk samples. Using an immunoenzymatic diagnostic kit, beta-lactam residues were detected in 17.0% of samples from Paraná, Brazil [11]. Using a method other than immunoenzymatic assay, 65.0% of the milk samples in Rio de Janeiro (Brazil) were contaminated with antibiotic residues [13]. Similarly, a commercial kit (Charm^®^ Cowside II Test) was used to determine the presence of antibiotics in 17% of pasteurized milk samples [12]. To the best of our knowledge, this is the first report of antibiotic residues in pasteurized whole milk intended for human consumption in Colombia.

A meta-analysis showed antibiotic residues in pasteurized milk in approximately 21% of the cases in Iran [9]. In 2015, a study was conducted in Burkina Faso, Africa, to analyze the presence of antibiotic residues in different dairy products. We found that 2.4% of pasteurized milk samples contained aminoglycoside and quinolone residues, while 66.7% contained beta-lactam, sulfonamide, and tetracycline residues. This result indicates poor sanitary control in the dairy production system [8]. Overall, these studies show antibiotic residues in pasteurized milk as a common finding worldwide and highlight the need for major surveillance and control in the production and marketing of milk. There is a need to ensure the absence of antibiotic residues in milk and other dairy products by controlling and monitoring the use of antibiotics in animals and in the dairy industry [35].

Of the 102 milk samples analyzed, 6% tested positive for beta-lactams within 30% of the milk brands. Tests were performed using an immunoenzymatic commercial kit that meets the FDA and Food Codex standards for quick detection of antibiotic residues. Our findings indicate non-compliance with both international and national regulations regarding antibiotic residues in milk [15, 16].

The presence of antibiotic residues in milk and milk microbial contamination with mesophilic bacteria and coliforms is a concern for public health. These contaminants may be harmful to consumers, especially vulnerable populations. Antibiotic residues may contribute to the development of antibiotic-resistant bacteria, making it more difficult to treat infections in animals and humans. The number of aerobic mesophilic bacteria and coliforms (both fecal and total) is an indicator of food hygiene and quality and the presence of other pathogens may indicate the presence of other pathogens at higher levels. This study demonstrates the need for strong control and surveillance of the dairy industry process in the country to ensure food safety and prevent the presence of these contaminants in milk.

## Conclusion

This study demonstrated the presence of antibiotic residues and microbial contamination in commercially available pasteurized whole milk intended for human consumption in Medellín, Colombia. Future scope of the study should focus on monitoring the entire dairy production chain to detect critical control points in the production and marketing milk process associated with microbial contamination and antibiotic residues. The present study was not developed in conjunction with government health control entities; this is the greatest limitation of the study because the results cannot be used to improve public policy to improve control in the dairy industry and ensure the quality of milk intended for human consumption.
